# Blockade of *Plasmodium falciparum* erythrocyte invasion: New assessment of anti-*Plasmodium falciparum* reticulocyte-binding protein homolog 5 antibodies

**DOI:** 10.3892/etm.2015.2237

**Published:** 2015-01-29

**Authors:** YAN SHEN, JUN WANG, XUEWU LIU, JIAO LIANG, YUXIAO HUANG, ZHONGXIANG LIU, YA ZHAO, YINGHUI LI

**Affiliations:** Department of Medical Microbiology and Parasitology, The Fourth Military Medical University, Xi’an, Shaanxi 710032, P.R. China

**Keywords:** *Plasmodium falciparum*, vaccine, invasion

## Abstract

There is great interest in any new discoveries in malaria vaccine research. *Plasmodium falciparum* reticulocyte-binding protein homolog 5 (PfRH5) shows promise in this area and may be used together with other merozoite antigens as a potential vaccine. In the present study, a bioinformatics prediction approach was applied to a PfRH5 B-cell epitope, and two B-cell epitope distributions were selected. Antibodies against the two PfRH5 distributions were obtained and the growth activity inhibition was measured. No inhibition of the *P. falciparum* CY strain was found, but the growth of the *P. falciparum* 3D7 strain was inhibited by all of the antibodies, in contrast to the results of other studies. It was additionally found that certain quantities of protein led to the inhibition of the parasitic invasion. Equally noteworthy was that the survival time of the group immunized with a portion of PfRH5 was significantly longer than that of the group immunized with the full-length protein, following infection by *P. berghei* ANKA. The present study produced conflicting results in *in vitro* and *in vivo* experiments, although the accuracy of the evaluation may be lessened due to the use of a murine malaria model. The findings of the present study may indicate that PfRH5 may not be suitable in malaria vaccine research.

## Introduction

Malaria remains one of the most serious infectious diseases of the 21st century. The development of an 80% efficacious malaria vaccine by 2025, which could offer protection for ≥4 years, is a worldwide goal. RTS,S/AS01 is raising the bar in the development and approval of a first-generation malaria vaccine that has >50% protective efficacy against severe disease and mortality and lasts longer than one year. RTS,S/AS01 is based on the major *Plasmodium* circumsporozoite surface protein and has reached phase III clinical trials; however, it is known that this vaccine does not confer complete sterile protection (1).

The parasite invasion process involves the duffy binding-like protein family (2,3) and *Plasmodium falciparum* reticulocyte-binding protein homologs (PfRHs) (4). Among these proteins, PfRH5 is vital for erythrocyte invasion (5) and has become a promising vaccine target (6). The protein basigin has been identified as the erythrocyte receptor of PfRH5 and is essential for the invasion of multiple strains of the pathogen (7,8). In a previous study, PfRH5 had an effect on several strains of *P. falciparum*, yet an antibody of a part of PfRH5 was considered not to inhibit the *P. falciparum* invasion (6). Despite this, a small fragment may be more suitable for vaccine design. Due to the lack of a suitable animal model (9), the *in vivo* immune effects of partial and full-length fragments of PfRH5 remain unknown. The present study established a rodent model to evaluate the effects of *P. falciparum.*

## Materials and methods

### Animals and parasites

The protocols in this study were approved by Xiking Hospital Medical Ethics Committee of the Fourth Military Medical University (Xi’an, China). The animals were well cared for and immunized using narcotic drug treatment to alleviate the pain. The female BALB/c mice were obtained from the Experimental Animal Center of the Fourth Military Medical University. A total of 40 mice used in the experiment were sacrificed by cervical vertebra dislocation. Cryopreserved *P. berghei* ANKA parasites (maintained in our laboratory) were thawed and passaged once *in vivo* prior to being used to infect the experimental animals. The *P. falciparum* CY and 3D7 strains (maintained in our laboratory) were cultured with human blood cells in RPMI-1640 medium containing 10% human sera as designed by Trager and Jensen (9). Two rounds of sorbitol treatment were used to synchronize the asexual stages as described previously (10).

### Prediction of B-cell epitopes of PfRH5

Bioinformatics methods, including Jameson-Wolf (11), Garnier-Robson (12), Chou-Fasman (13) and Karplus-Schulz (14), were used to predict the signal peptides, transmembrane domains, hydrophobicity, secondary and tertiary structures, potential B-cell epitopes and other properties of the B-cell epitope of PfRH5. The ability to predict the B-cell epitopes of PfRH5 provided a basis for the preparation of the PfRH5 vaccines.

### Expression and purification of recombinant (r)PfRH5 protein

Antigenic prediction showed that the highest score of the linear epitope region of PfRH5 was concentrated at amino acid positions 200–400, suggesting that these amino acids may play an important role in the function of PfRH5; therefore, the corresponding DNA sequences for amino acids 200–300 (PfRH5-23), 300–400 (PfRH5-34) and the full-length amino acid sequence (PfRH5-FL) were cloned into the prokaryotic expression vector pET32a(+) (Novagen, Darmstadt, Germany) with *Bam*HI and *Hin*dIII sites. The nucleic acid sequences of interest were codon optimized to permit expression in *Escherichia coli*. Recombinant proteins were induced in the *E. coli* BL21 strain (maintained in our laboratory) by the addition of 1 mM isopropyl β-D-1-thiogalactopyranoside (IPTG) for 4 h. The fusion protein was isolated by nitrilotriacetic acid (NTA) affinity chromatography. PfRH5-34 and PfRH5-FL were expressed in the inclusion body of the insoluble protein. Protein isolated from the inclusion bodies was refolded and purified on Ni^2+^-NTA agarose resin (Qiagen, Valencia, CA, USA) under native conditions. The expression and purification of recombinant proteins was confirmed by western blotting with His tag antibody (mouse monoclonal; 1:1,000; Beyotime Institute of Biotechnology, Shanghai, China).

### Antibody generation

Immunizations were conducted in eight-week-old BALB/c female mice. Mice were immunized three times on days 0, 14, 28 and 40 by intraperitoneal injection of 20 *μ*g fusion protein (PfRH5-23, PfRH5-34 or PfRH5-FL) and an equal volume of Freund’s adju vant (F5881; Sigma-Aldrich, St. Louis, MO, USA). The mice sera were collected on day 40 and used in all of the following experiments.

### ELISA and indirect immunofluorescent assay (IFA)

Anti-PfRH5 specific antibody titers in the sera were determined using a standard ELISA procedure using rPfRH5 (prepared by our department) (15). ELISA was performed three times on each serum sample in 96-well polyvinylchloride microtiter plates. The plates were coated with 0.2 *μ*g/well purified rPfRH5 and left overnight at 4°C. Following the coating, and between each incubation step, the plates were washed three times with phosphate-buffered saline-Tween 20 (PBS-T). The plates were then blocked with 4% fat milk in PBS-T for 1 h at 37°C. Diluted serum samples were added to individual wells and incubated for 1 h. Subsequent to washing, 100 *μ*l horseradish peroxidase-conjugated polyclonal sheep anti-mouse immunoglobulin G (IgG) (1:5,000; Beyotime Institute of Biotechnology) was added and the mixture was incubated for 1 h at 37°C. Color development was performed by the addition of 100 *μ*l/well 3,3,5,5-tetramethylbenzidine (Sigma, St Louis, MO, USA). Absorbance was read at 450 nm after 30 min using a plate reader (Model-680; Bio-Rad, Hercules, CA, USA).

### Giemsa stain

The thick blood film was left to dry in an incubator at 37°C for 1 h and a fresh 4–5% Giemsa solution was prepared in PBS (pH 7.1). The slides were placed on a staining rack and 1 ml Giemsa solution (G4507; Sigma) was added onto the slides; the slides were subsequently stained for 20 min. Following gentle rinsing, the slides were left to dry in an upright position and were observed under a microscope (magnification, ×100; Olympus BX51; Olympus Corporation, Tokyo, Japan) using immersion oil (BA-7003A; Baso Diagnostics Inc., Zhuhai, China).

### Western blot analysis

A total of ~10 μg recombinant PfRH5-FL, PfRH5-23 and PfRH5-34 were separated on a 15% SDS-PAGE gel. Following electrophoresis, proteins were transferred to a nitrocellulose membrane. After transfer, the membrane was blocked with 5% milk in PBS-T for 1 h at room temperature and washed three times with PBS-T. Membranes were incubated overnight at 4°C with a His-tagged monoclonal mouse antibody (1:1,000 dilution in PBS-T; Beyotime Institute of Biotechnology). Following washing three times with PBS-T, the membranes were incubated with an HRP-conjugated polyclonal goat anti-mouse antibody (1:5,000 dilution in PBS-T; Beyotime Institute of Biotechnology). Detection was carried out using an ECL kit (Millipore Corporation, Billerica, MA, USA).

### In vitro assay of P. falciparum invasion inhibition

The effect of mouse sera and rPfRH5 on the parasite invasion of human red blood cells (RBCs) was assessed in three experiments using an invasion inhibition assay. Synchronized trophozoites of the *P. falciparum* CY and 3D7 strains were purified by centrifugation at 1,500 × g and 4°C for 15 min on Percoll gradients (16). To obtain a final hematocrit of 2% and parasitemia of 0.2%, ~7.2×10^5^ schizonts in 180 ml complete medium with 10% human serum were mixed with 4×10^7^ human RBCs. Each well received 180 *μ*l of the culture and 20 *μ*l heat-inactivated (56°C, 30 min) mouse anti-PfRH5 sera (or rPfRH5 protein). The medium was changed every 24 h. After incubation for 72 h, the number of parasites was determined; the parasites were collected by centrifugation (3 min at 1,000 × g) and the supernatant was removed by aspiration (17). Hoechst 33258 stain (no. 861405; Sigma-Aldrich) in PBS (0.1 ml) was added to each well to cover the complete surface of the well and left for 10 min at 37°C. The stain was removed by centrifugation and the RBCs were washed twice with PBS. Fluorescence intensity was read at 450 nm after 10 min using a GENios Pro Reader (Tecan, Männedorf, Switzerland). The percent inhibition was calculated using the fluorescence intensity data in the pre-immunization serum controls as 100% invasion.

### In vivo assay of P. berghei ANKA invasion inhibition

Mice were immunized three times on day 40 by intraperitoneal injection of 20 *μ*g fusion protein (PfRH5-23, PfRH5-34 or PfRH5-FL) and an equal volume of Freund’s adjuvant. The mice were challenged by 1×10^5^ *P. berghei* ANKA RBCs with parasitemia of 5%. The survival time and parasitemia levels of the mice were observed every day.

## Results

### B-cell epitope prediction, expression and purification of rPfRH5

**Figure 1:**
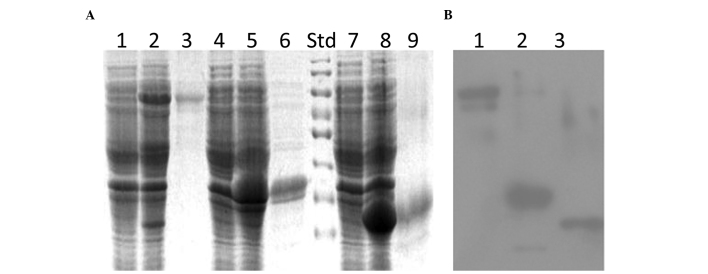
Production of recombinant PfRH5. (A) Coomassie blue-stained sodium dodecyl sulfate-polyacrylamide gel electrophoresis gel showing rPfRH5 fragment expression in *Escherichia coli* and their purification by Ni^2+^-NTA-agarose. Lanes 1, 4, and 7, uninduced; lanes 2, 5 and 8, rPfRH5-FL, rPfRH5-34 and rPfRH5-23 induced with 1 mM isopropyl β-D-1-thiogalactopyranoside, respectively; lanes 3, 6 and 9, rPfRH5-FL, rPfRH5-34 and rPfRH5-23 protein purified under native or denaturing conditions by Ni^2+^-NTA-agarose, respectively. (B) Detection of rPfRH5 expression. Lanes 1 (rPfRH5-FL), 2 (rPfRH5-34) and 3 (rPfRH5-23), mouse anti-His antibody. Std, protein standard; NTA, nitrilotriacetic acid; rPfRH5, recombinant *Plasmodium falciparum* reticulocyte-binding protein homolog 5.

The sequence of PfRH5 from the *P. falciparum* 3D7 strain has been reported previously (GenBank accession no. XM_001351508). Prediction of the B-cell epitope for the PfRH5 antigen is useful for the preparation of PfRH5 antibodies using a synthetic peptide approach or for fusion with another protein. Screening for the B-cell epitope of the PfRH5 antigen with bioinformatics revealed that an abundance of the B-cell epitope was located at amino acids 200–400. The DNA sequences for PfRH5-23, PfRH5-34 and PfRH5-FL were optimized to permit expression in *E. coli*. rPfRH5 expression and purification were observed subsequent to IPTG induction (Fig. 1A). The PfRH5-34 proteins detected with sodium dodecyl sulfate-polyacrylamide gel electrophoresis were slightly larger than the expected size. Their diffuse appearance may indicate misfolding of the rPfRH5 in the *E. coli* cells. The recombinant PfRH5 bands were also detected using anti-His tag antibody (Fig. 1B).

### rPfRH5 vaccination

**Figure 2:**
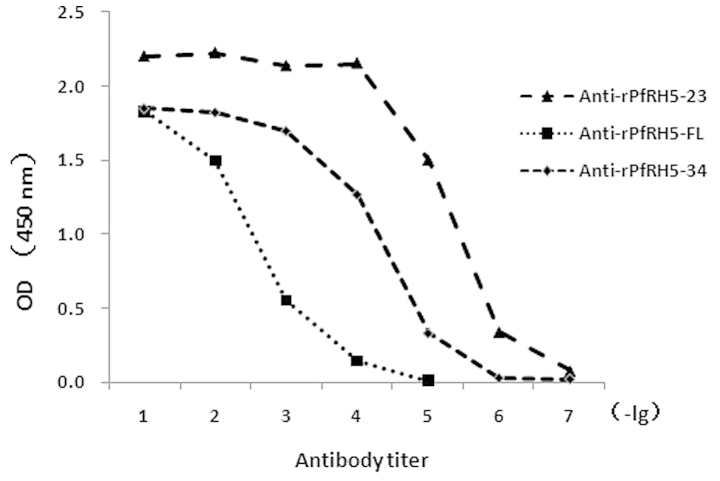
Production antibodies of rPfRH5. rPfRH5-specific antibody levels were detected in the immune sera and expressed as values at OD 450 nm. OD, optical density; rPfRH5, recombinant *Plasmodium falciparum* reticulocyte-binding protein homolog 5.

PfRH5-specific antibody titers increased over the entire immunization course, and the final titers on day 40 reached 10^4–^10^6^ (Fig. 2). In addition, thin blood smears of *P. falciparum*-infected RBCs were prepared for examination of the antibody specificity against PfRH5 by IFA. Polyclonal anti-rPfRH5 antibodies (1:1,000) generated from mice were used as primary antibodies. Following washing with PBS, the cells were incubated with fluorescein isothiocyanate-conjugated anti-mouse IgG antibody (Sigma). Immunofluorescence was visualized using fluorescence microscopy (Olympus BX51; Olympus Corporation). Bright fluorescence was observed when merozoites were released and contained in mature schizonts using the rPfRH5 immune sera, whereas pre-immunization sera showed only background fluorescence. These data suggested that antibodies against rPfRH5 could detect native epitopes on PfRH5 (data not shown).

### Easy detection of parasitemia

**Figure 3:**
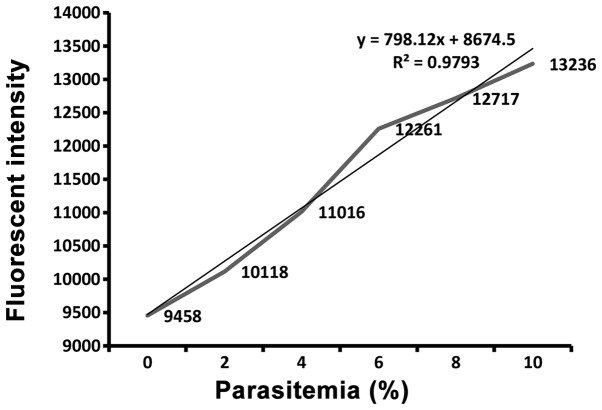
Relevance of fluorescent intensity and microscopy of thin blood smear counting. The parasitemia standard was determined by microscopic examination of blood smears. Thin blood smears were prepared on glass slides and fixed with 100% methanol. The smears were stained with a filtered 1/10 dilution of Giemsa solution in phosphate-buffered saline, incubated for 20 min at room temperature, washed with distilled water, dried and observed under an oil immersion lens (magnification, ×100). Parasitemia was determined by the counting of ~2,000 erythrocytes.

Fluorescence intensity-based evaluation of Hoechst 33258-stained parasites correlated well with the microscopic counts of the Giemsa-stained smears (Fig. 3). The relevance of the Hoechst 33258 stain-based fluorescence intensity method for detecting the parasite infection rates was tested. The consistency of detection using fluorescence intensity and counting under an oil immersion lens (18) was good.

### Inhibition of parasite invasion

**Figure 4:**
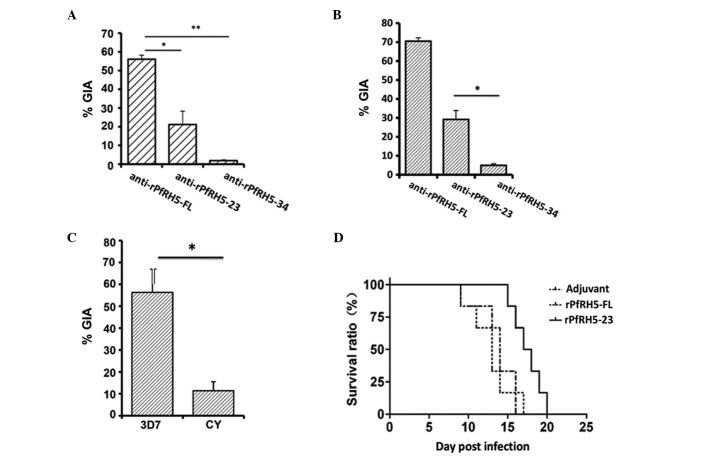
Inhibition of parasite invasion with anti-rPfRH5 IgG or rPfRH5 protein *in vitro* and *in vivo*. (A) Percentage growth inhibition against the 3D7 parasite clone with IgG from mice immunized with rPfRH5-FL, rPfRH5-23 or rPfRH5-34. (B) Percentage growth inhibition against the 3D7 parasite clone with rPfRH5-FL, rPfRH5-23 or rPfRH5-34 at a protein concentration of 0.1 nM. (C) Percentage growth inhibition against the 3D7 parasite and the CY parasite with IgG from mice immunized with rPfRH5-FL. (D) Survival rate and time in a murine malaria model immunized with rPfRH5-FL, rPfRH5-23 or rPfRH5-34. Data represent the mean of triplicates from two independent experiments. Bars indicate the standard error of the mean for the six replicates over two experiments. Asterisks indicate the predicted and observed values that differed significantly (two-way analysis of variance with Bonferroni post-hoc testing). rPfRH5, recombinant reticulocyte-binding protein homolog 5; rPfRH5-FL, recombinant PfRH5-full length amino acid; rPfRH5-23, rPfRH5-amino acids 200–300; rPfRH5-34, rPfRH5-amino acids 300–400; IgG, immunoglobulin; GIA, growth inhibition activity.

In order to determine the effect of the immune sera on merozoite invasion inhibition, schizonts from the *P. falciparum* 3D7 strain were purified and used in an RBC invasion assay. The results showed that antisera from the PfRH5-FL group more effectively inhibited the merozoite invasion than antisera from the PfRH5-23 and PfRH5-34 groups (Fig. 4A), suggesting that the *in vitro* merozoite invasion inhibition effect is potentially associated with the PfRH5-FL antigen. Of note, the PfRH5-23 was more effective in merozoite invasion inhibition than the control protein rPfRH-34 (Fig. 4B), and antisera from the PfRH5-FL group were not found to be effective in the RBC invasion assay for the *P. falciparum* CY strain (Fig. 4C). The mice were challenged by *P. berghei* ANKA. The parasitemia of the PfRH5-34 and PfRH5-FL groups was higher than that of the control (data not shown), while the parasitemia of the PfRH5-23 group was lower than that of the control. Furthermore, the survival time of the PfRH5-23 group was longer than that of any other group, including the control, suggesting that any *in vivo* merozoite invasion inhibition effect is likely to be associated with the PfRH5-23 antigen (Fig. 4D).

## Discussion

Vaccination against malaria is generally presumed to be the most cost-effective way of protecting against the disease, and it is hoped that it can potentially eradicate malaria from the world. Studies (19–21) have shown different transcription and expression patterns for RH genes in several *P. falciparum* parasite strains and isolates. PfRH5 does not change its expression levels in *P. falciparum* strains among different invasion routes and it is recognized in sera from patients suffering from natural malarial infection (15), which suggests that it is an excellent candidate for inclusion as a component in a fully effective anti-malarial vaccine. Reports published during the past decade (15,22,23) have shown that PfRH5 fragments bind to receptors on the RBC membrane.

In the present study, antibodies against PfRH5 and PfRH5 fragments were obtained in mice for the evaluation of *P. falciparum* 3D7 and CY strain schizont inhibition. High titers of PfRH5 and PfRH5 fragment antibodies that specifically recognized PfRH5 in late-stage schizonts were revealed by IFAs similar to those reported for other RH family members (data not shown) (24–26). Studies have shown the crucial role of PfRH5 in RBC invasion due to the association between PfRH5 and basigin (7,8), suggesting that PfRH5 may be a candidate for inclusion in a fully effective anti-malarial vaccine. In this light, recent studies (27–30) showing that vaccine-induced anti-PfRH5 antibodies can potently inhibit different parasite strain invasions *in vitro* are encouraging and suggest that future malaria vaccine research may focus heavily on this basigin-PfRH5 interaction.

In the present study, however, contradictory results were obtained: i) Inhibition of the parasite invasion was not observed in the *P. falciparum* CY strain; ii) the PfRH5-23 protein could prolong survival time *in vivo*, but the PfRH5-FL protein did not exhibit the same results, despite having the ability to inhibit parasite invasion *in vitro*. Although studies in mice do not meet the evaluation criteria for human *P. falciparum* vaccines, the murine malaria model is useful. Furthermore, *in vitro* studies of vaccine-induced mouse antibodies are easier to conduct than *in vivo* studies. Whereas known quantities of antibodies in studies *in vitro* can be administered, the quantity and specificity of animal antibodies produced against each vaccine cannot be controlled *in vivo* and may vary substantially between individuals. In conclusion, PfRH5-FL may not be as promising as was hoped. By blocking parasite invasion through PfRH5, the vaccine appears to be some distance away from being able to halt malaria. The identification of basigin, a well-characterized membrane protein, as a receptor that is essential for malaria infection, is likely to contribute significantly to the prevention and treatment of malaria.
